# The Relationship between Knowing and Liking for 91 Urban Animal Species among Students

**DOI:** 10.3390/ani13030488

**Published:** 2023-01-31

**Authors:** Fabio S. T. Sweet, Peter Noack, Thomas E. Hauck, Wolfgang W. Weisser

**Affiliations:** 1 Terrestrial Ecology Research Group, Department of Life Science Systems, School of Life Sciences, Technical University of Munich, 85354 Freising, Germany; 2 Department of Educational Psychology, Institute of Psychology, University of Jena, 07743 Jena, Germany; 3 Department of Landscape Architecture and Planning, Institute for Urban Design and Landscape Architecture, Vienna University of Technology, 1040 Vienna, Austria

**Keywords:** urban ecology, animal preferences, animal familiarity, socio-ecology, Germany

## Abstract

**Simple Summary:**

People in cities have varying opinions about the animals around them, but there is rather little knowledge on how attitudes towards different urban animals compare with each other and how that relates to how familiar they are with them. Using self-reporting questionnaires, we found that students thought themselves familiar with most animals, and that the animals were not equally liked. While the more familiar animals were not also the better liked ones, we did find that attitudes towards familiar animals were more extreme on both the positive and negative sides. We suggest that more research in which drivers of attitudes towards many different animals can be compared is needed to reduce conflict between animal and human urban inhabitants.

**Abstract:**

While there is growing consensus that nature should be promoted in cities, it is less clear what kind of nature this should be. One hypothesis is that humans show greater liking for those parts of nature that they know better. Using questionnaires, we studied the familiarity of 475 students with 91 urban animal species and the relationship between familiarity and attitudes towards the species. Students declared that they were familiar with most animals, but not all animals were equally liked. Better-known species were not generally the better-liked ones. The more familiar animal species were the more extreme attitudes became towards them, both positively and negatively. Our research shows that familiarity and attitude are not two sides of the same coin. It also emphasizes that there are parts of nature that are not liked by many humans and that this dislike is not necessarily correlated with insufficient knowledge. Detailed studies of what components of nature humans like and reasons underlying this are necessary to successfully increase nature in cities.

## 1. Introduction

Urban nature is important for city dwellers. This is because most humans now live in cities, which makes cities the prime place where humans can experience nature in their day-to-day life. There is increasing evidence that urban nature has positive effects on human well-being and health [1,2], and city dwellers also profit from other ecosystem services provided by urban nature [3,4,5,6]. Today, there is widespread agreement that urban nature is valuable and needs to be saved and fostered [4,7,8,9,10]. However, while there is a general consensus that nature should be increased in cities, it is less clear what kind of nature should be targeted. This is not only because humans differ in their attitudes to nature [11,12], but also because there are elements of nature, e.g., particular species, that are more liked than others [13,14,15]. Because human attitudes will affect the acceptance of programmes to conserve or enhance nature, it is important to investigate people’s relations to components of nature [16]. Here, we focus on human attitudes towards animals living in cities, because animals are an important component of urban nature that humans perceive.

A large number of studies have asked humans about their attitudes towards particular species, although the purpose of the studies differed greatly. One set of studies specifically focusses on wildlife conflicts that often concern large mammals which can potentially be dangerous to either humans themselves or to farm animals. These conflicts can concern wildlife coming to cities, as in the case of coyotes, which are medium-sized predators that can thrive in cities in the US and cause conflict through pet killings and attacks on humans [17]. They can also concern wildlife only outside cities or inside and outside cities, such as the wolves in Europe [18], bears in Europe or the US [19,20] or species of the Serengeti National Park in Tanzania [21]. There are also a number of studies that investigated the attitudes of humans to a larger range of species, up to 33 [11,12,22,23,24,25,26,27,28,29,30,31,32,33,34]. These studies found strong differences in attitudes towards different animal species, from a clear dislike (e.g., spiders) to very positive attitudes (e.g., squirrels).

Closely related to the question of what animals humans like is whether the attitudes towards a species, or a group of species, depends on how familiar humans are with these species. Several studies report that contact with nature can increase people’s tolerance towards nature and willingness to protect it [11,33,35,36,37,38]. The idea that more familiarity will improve attitudes is also the basis of many environmental education programmes [39,40]. In the theory of environmental education, knowledge affects both attitudes and action [41]. Environmental education aims at increasing knowledge about the environment, e.g., about animals, which is then assumed to lead to more positive attitudes, and to more pro-environmental behaviour, e.g., active support of species conservation [42]. Environmental education has frequently been shown to increase the knowledge of participants, but the effects on a change in attitudes, or even increased pro-environmental behaviour, are less clear [40,43]). While educational programmes and public information have been able to positively affect attitudes towards generally disliked animals such as crocodiles, snakes and tarantulas (reviewed in 25), there are only a few studies that test whether attitudes towards particular urban animal species are positively related to familiarity with a species [12]. On the other hand, it has been postulated that a lack of knowledge can be a barrier to behavioural change, such that knowledge is a necessary but not sufficient condition for behavioural change [44]. Interestingly, people who express pro-environmental attitudes do not necessarily have better environmental knowledge than those who have less positive attitudes towards the environment [45]. Both knowledge and liking can also arise through the “mere exposure” effect, coined by Zajonc in [46], whereby repeated exposure to stimuli enhances attitudes towards such stimuli, which has since been affirmed by many studies across a wide range of disciplines and objects, including the marketing of products [47]. Importantly, there is also evidence that repeated exposure to a negatively viewed stimulus can weaken the positive effect [48] or strengthen a negative sentiment [49]. Some evidence that this is also true for attitudes towards nature comes from studies comparing the attitudes of people from rural areas to those from cities. For example, [22] found that children from the city generally like animals more than those from rural areas and [18] found that people that have grown up in a household with farm animals have more negative attitudes towards wolves. In general, however, the relationship between knowing an animal and attitudes towards it has not been investigated in much detail or for many species.

In this study, we explored the relationship between knowing and liking for a large number of animal species. We asked university students in Germany to report on their familiarity with 91 species or groups of animal species and also asked for their attitudes towards these animals. We addressed the following questions:

1. To what degree are the different animal species known and liked by students?
2. Are the better-known species also the more liked ones?

## 2. Materials and Methods

A survey was conducted with the use of an online questionnaire made through SoSciSurvey [50]. A questionnaire was considered to be an appropriate method because it limits the range of answers a participant can give and allows for a standardization of results. We used a Likert scale to assess levels of familiarity and liking for each species.

Surveys were conducted at the Technical University of Munich, the University of Jena and the University of Kassel. Participants were informed beforehand about the goal of the questionnaire and could voluntarily join the questionnaire by following a link or a QR code. Participants were also free to finish answering the questionnaire at any point, after which their answers would not be taken into consideration. Our procedure assured full anonymity since it was not possible to find out who participated in the questionnaire. We also encouraged students to share the questionnaire with other people they knew. The survey took place from 22 October 2019 to 12 February 2020.

### 2.1. Questionnaire

The questionnaire had 188 questions. Six of these were demographic questions, of which two were optional; 91 of these related to familiarity with animals; and 91 of these related to attitudes towards animals. The biologists of the team drew up a list of potential species and then the social scientists and planners of the team went through the list to trim it to below 100. We deliberately kept some species that many people may not have been familiar with but which are sometimes closely associated with human housing (e.g., dormice). We chose 91 animal species or species groups among mammals, birds, reptiles, amphibians, arthropods and other invertebrates in an attempt to cover a wide range of species (Table A1). These animals were pre-tested with a smaller group of students. Taxa that were difficult to distinguish for non-experts, due to similarity or small size, were grouped at the genus level (e.g., ‘redstarts’, and ‘dormice’) or at an even higher taxonomic level (e.g., spiders). For the sake of conciseness and consistency, these will henceforth still be referred to as “species”. To be able to account for sociodemographic differences between participants, and account for the variable quantity of participants from different sociodemographic backgrounds, basic classifying questions were asked with regard to participants’ gender, age, formal education, profession, region of residence and country of origin. Participants were not obliged to disclose their country of origin, and participants that were not residents of Germany were not obliged to disclose their region of residence. The questionnaire could be answered in either German or English. We presented the common name of each animal in the selected language of the questionnaire. There were no pictures of animals provided with the questionnaire The questionnaire was on average finished in 9 min and 20 s (±4 min).

### 2.2. Measures

There were five response options for attitudes towards animals, with the most negative being “very disliked” (score = 0), the middle option being “Neutral” (score = 2) and the most positive option being “very liked” (score = 4), similar to previous research [12,26]. Familiarity with the animal was divided into three response options. In addition to the answers ‘I do not know it’ (score = 0), and ‘I know and I would recognize it’ (score = 2), we added ‘I know it, but I would not be able to recognize it’ (score = 1), as an indicator of intermediate familiarity. The latter option was added because people might have, for example, heard of or read about a certain animal, which potentially influences their attitudes, without being able to recognize it when presented with it.

### 2.3. Data Management

All questionnaire answers were imported directly from the SoSciSurvey server into R [51]. Scripts were written in RMarkdown [52,53] and in RStudio [54], and composite figures were composed with the patchwork package [55]. Summary statistics were calculated with the summarySE function of the Rmisc package [56].

### 2.4. Participants

**Figure A1:**
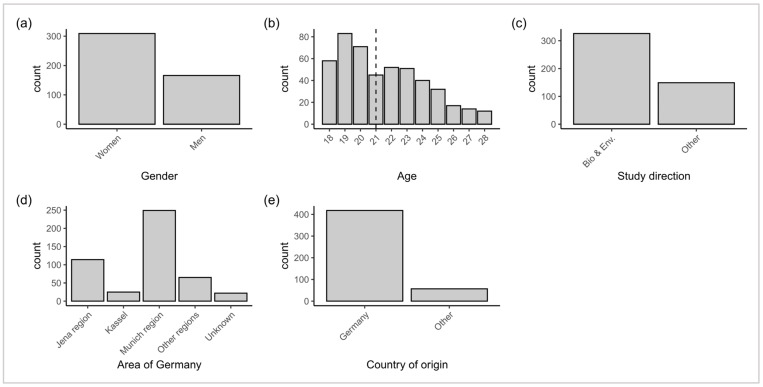
Distribution of demographic groups of participants: (**a**) gender, (**b**) age, (**c**) study direction, (**d**) Area of Germany, (**e**) country of origin.

A total of 836 questionnaires were started. Of those questionnaires, 666 were completed. 191 were removed because they were not completed by students, because students did not state their field of study in a way that could be deduced from the response or because the respondents were younger than 18 or older than 28. Responses from participants over 28 were discarded because of the low number of observations recorded from participants above that age. The final dataset comprised completed questionnaires of 475 participants. Of these 475 participants, 309 self-reported as female, while 166 self-reported as male; 326 studied biology or environment-related studies, and 149 studied in other fields; 114 were from the Jena region, 249 from the Munich region, 25 from Kassel, 65 from other regions in Germany, and for 22 their home region was unknown; 418 were from Germany, and 57 were foreign students in Germany. The age distribution was skewed towards the younger age groups, with a median age of 21 years (see Appendix B, Figure A1).

### 2.5. Statistical Analysis

All statistical analysis was done using R [51]. We conducted multiple types of analyses at the level of species but also at the level of higher taxa.

#### 2.5.1. Familiarity and Attitudes across Species

We performed two-sided Bonferroni-corrected t-tests to test for each species whether the means of participants’ familiarity with, and attitudes towards, the species were significantly higher or lower than “neutral” (e.g., “1” in the question of familiarity, “2” in the question of attitudes). The same analysis was applied to higher taxa. Additionally, we performed pairwise comparisons between the higher taxa.

#### 2.5.2. Relationship between Familiarity and Attitudes

Correlation analyses were used to investigate relationships between familiarity and attitudes across species (irrespective of individual participants).

The mean familiarity of and mean attitude towards each species was used to investigate whether there was a correlation between how familiar an animal species was and how well it was liked. In order to investigate whether the spread of familiarity or attitudes were influenced by their respective means, standard deviations of familiarity and attitude for each species were related to the respective means of those species’ familiarity and attitude.

In order to test for a relationship between the spread of attitudes towards species and familiarity with them, the mean value for attitudes towards species was calculated for values within five equidistant bins on familiarity. In order to test whether the mean attitude towards animal species became more extreme (either positively or negatively) with increasing familiarity, the attitude scale was centered around zero (by subtracting 2 from all values) and the absolute value was taken to test how absolute attitude value depends on familiarity.

To test if people who know a species better also have more positive attitudes towards the species we conducted Pearson’s correlation coefficient tests between familiarity and attitudes for each species separately, using participants as a replicate. The same analysis was also done at the higher taxonomic levels.

## 3. Results

### 3.1. Familiarity and Attitudes across Animal Species

**Figure 1:**
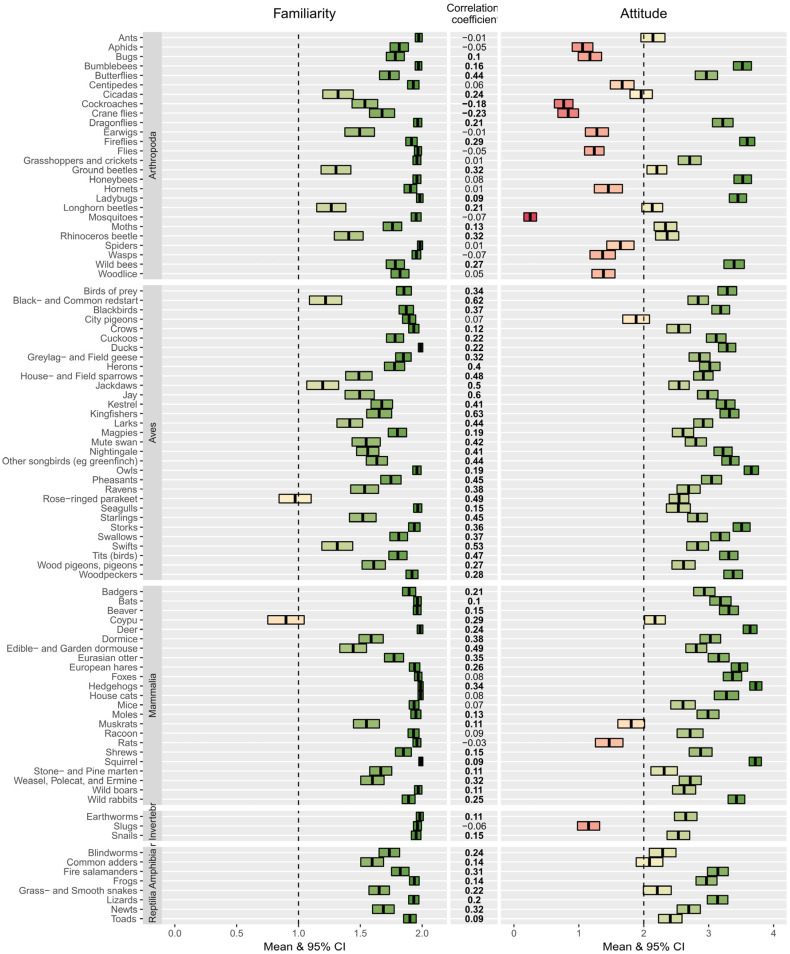
Mean familiarity and attitude towards individual animal species and the correlation coefficient between them. Boxes indicate mean ± 95% CI. Bold correlation coefficient values indicate a significant correlation; cursive values indicate no significant correlation. See Appendix C for a tabular representation of the data.

Participants of the questionnaire reported that most animals in the questionnaire were familiar. Mean familiarity was 1.75 ± 0.05 (CI), significantly greater than one (t90 = 29.34, *p* < 0.001). For 90 out of the 91 species, individual mean familiarity was greater than one at *p* < 0.0005 (Bonferroni corrected *p*-value for 91 species tests 0.05/91, Figure 1). The exception was the rose-ringed parakeet. Students were most familiar with squirrels, ducks, hedgehogs and house cats.

The participants of the questionnaire had, on average, positive attitudes towards the animals presented. Mean attitude was 2.64 ± 0.16, significantly greater than two, i.e., neutrality (t90 = 7.92, *p* < 0.001). However, there were clear differences between species. A total of 73 species were liked better than neutral, 16 animals were on average disliked, and for two species, cicadas and common adders, mean attitude was not different from neutral (Figure 1). Within the group of arthropods there was a high variability in mean attitudes between the different species in this group. The most notably disliked arthropod species were mosquitoes and cockroaches, and the most notably well-liked arthropods were bees and fireflies.

### 3.2. Relationship between Familiarity and Attitudes

**Figure 2:**
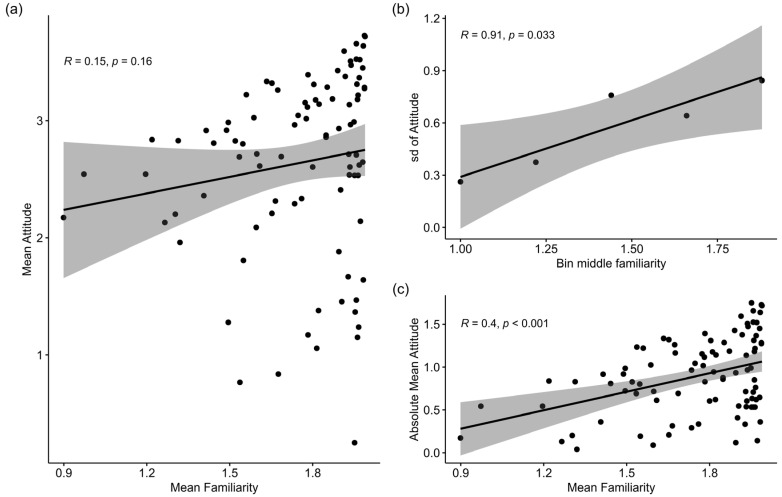
Across-species patterns in participants’ attitudes towards and familiarity with animal species. (**a**) Correlation between the average scores of familiarity and attitude, calculated for each species across all participants (*N* = 475). Each point represents a species. (**b**) Correlation between the standard deviation of attitudes to mean familiarity. Species were allocated to one of five bins based on their familiarity scores, and the standard deviation of attitudes was then calculated for all animal species in the bin (see Appendix D, Figure A2). (**c**) Correlation between mean familiarity and the absolute value of attitude, calculated by subtracting two from the mean scores and taking the absolute value. The grey areas around the lines represent the confidence interval.

There was no significant correlation between how familiar a species was and what the attitudes were toward the species (r_89_ = 0.15, *p* = 0.16; Figure 2a). However, the variability of attitudes towards species increased as familiarity increased (r_3_ = 0.91, *p* = 0.033; Figure 2b), i.e., for species people were more familiar with the attitudes of people differed more among the species. Additionally, the mean attitudes towards species became significantly more extreme (deviation from neutral) with increasing mean familiarity in both the positive and negative directions, i.e., the most liked and disliked species were among the most familiar species (r_89_ = 0.4, *p* < 0.001; Figure 2c).

**Figure A2:**
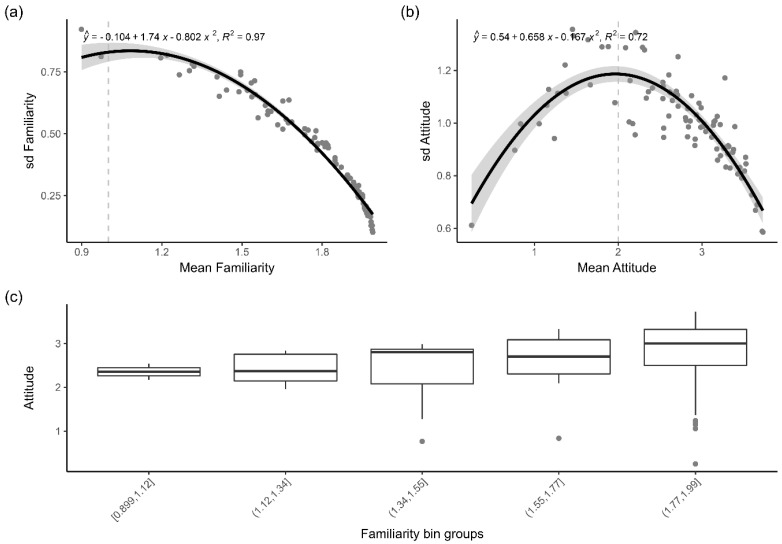
Relationship between variability in familiarity or attitude and the mean of the other variable. Each point in (**a**–**c**) represents a species and means, and standard deviations are calculated across participants. (**a**) Variability in familiarity (standard deviation) against mean attitude. (**b**) Variability in opinion (standard deviation) against mean familiarity. The dashed vertical line shows the middle value in the questionnaire. The grey areas around the lines represent the standard error. (**c**) Attitude as a function of familiarity. Familiarity was divided into five groups of equal distance, and then boxplots were made to visualize the attitudes across species in those groups.

When standard deviations of attitudes and familiarity for each species (i.e., variability among people in their familiarity or attitudes towards the same species) were plotted against their own respective means, variability peaked at intermediate values (Appendix D, Figure A2a,b). This indicates that species with a more extreme mean attitude or familiarity also have a higher degree of consensus among participants towards the score than species with a neutral mean.

The relationship between attitudes and familiarity across participants was also assessed for each animal species individually by correlating scores for familiarity and attitudes separately for each species using each participant as a unit of replication. For 68 species, there was a positive correlation between being familiar with the species and the attitudes towards that species, while for 21 species, there was no such relationship (Figure 1). Thus, within species, there was for many species the expected increase in positive attitudes towards the species with increasing familiarity. For two species, however, cockroaches and crane flies, there was a significant negative correlation between familiarity and attitudes, i.e., the better the species was known, the more disliked it was. In addition, the strength of the relationship between familiarity and attitudes within species differed strongly among species (Figure 1). For example, the correlation coefficient for deer was only 0.24 (*p* < 0.001), while the correlation coefficient for kingfishers was 0.63 (*p* < 0.001).

### 3.3. Taxon-Level Analyses

**Figure A3:**
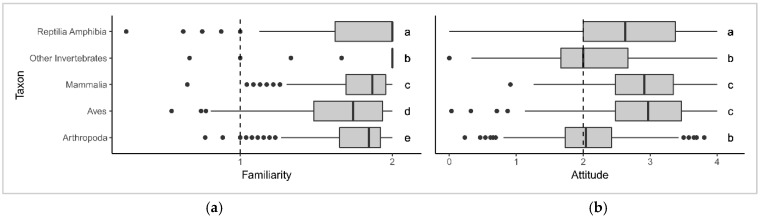
Familiarity ((**a**); F_4, 2370_ = 92.97, *p* < 0.001) and attitude ((**b**); F_4, 2370_ = 163, *p* < 0.001) at the taxon level. Taxa are alphabetically ordered. Equivalent letters behind the graph indicate groups that do not significantly differ from each other. *N* = 475.

**Figure A4:**
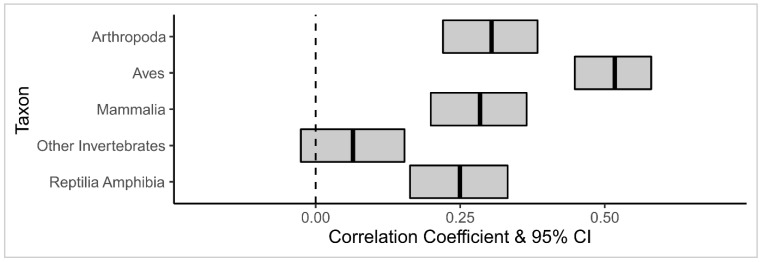
Correlations between attitudes towards and familiarity with each taxon. If the correlation coefficient is left of the dashed line at 0, that indicates a negative correlation; if the correlation coefficient is right of the dashed line at 0, that indicates a positive correlation. The boxes represent the estimated correlation coefficient ± 95% CI. *N* = 475.

Participants were quite familiar with the different taxa (Appendix E, Figure A3), reflecting the results of the species level. However, there were differences between taxa. Participants were most familiar with the group of ‘Other invertebrates’, which included slugs, earthworms and snails; this was followed by the equally familiar groups of amphibians/reptiles, mammals and arthropods; least familiar were birds, due to the fact that there were some species that were less known (Figure A3). Average attitudes towards the taxa were also generally positive, or at least neutral, with a notable divide in attitudes between the vertebrate groups, birds and mammals (more positive) and invertebrates, i.e., arthropods and other invertebrates (less positive or neutral, Figure A3). There was a significant positive correlation between the familiarity of participants with the taxon, i.e., pooled average of all species within a taxonomic group, and their attitudes towards the taxon, except for the group of “Other Invertebrates” (Appendix E, Figure A4).

## 4. Discussion

The goal of this research was to investigate the relationship between people’s familiarity with different animal species and their attitudes towards these species. Our study was carried out in a local context but included many animal species. Students were familiar, i.e., stated that they know of the animal, with a surprisingly high number of species. Students also had generally positive attitudes towards the majority of the species. Interestingly, there was no relationship between overall familiarity with a species and attitudes towards it; on the contrary, well-known species could also be very disliked. However, when species were analysed one by one, there was, for most animals, a positive relationship between individual participants’ familiarity with the species and the attitudes towards them.

### 4.1. Known and Unknown Species

Most of the species presented in our questionnaire are common in urban environments and students were on average quite familiar with them. The most familiar species were squirrels, ducks (as a group), hedgehogs and house cats, all of which are omnipresent in European urban environments, with house cats also being a common companion animal. Of the species people were least familiar with, the coypu is not as readily associated with urban environments, and the rose-ringed parakeet is a relatively recent alien addition to German cities. The result that students are less familiar with birds compared to other higher-level taxa is due to the fact that both the number of species as well as the taxonomic resolution were highest among birds. This lowered the average score for birds compared to other taxa such as arthropods, for which many higher-level taxa were included in the questionnaire. Thus, among birds, some species were not very well known, such as the rose-ringed parakeet, but also jackdaws, swifts or larks. The fact that many students reported that they are not familiar with swifts, a common bird in German cities, or with the common jackdaws and—in the countryside—larks, shows that the general knowledge of species of students is not high (similarly to [57]). It is also important to point out that our questionnaire relied on self-reported familiarity with species. Assessing familiarity with species in more detail requires the inclusion of questions on the biology of the species (e.g., in [58]). Because we aimed to include many animal species, we decided to rely on self-reported familiarity for this study.

Nevertheless, our study shows that participants are aware of the diversity of animals within cities, and suggests that the notion of a general ‘disconnect ‘ between people and nature [59,60,61] needs to be studied in more detail.

### 4.2. Liked and Disliked Species

Animals were clearly not all equally liked. For the higher taxa, birds, then mammals and then amphibians/reptiles were liked most, while attitudes towards arthropods and other invertebrates were less positive. As expected, species commonly categorized as ‘pests’ were not liked, such as cockroaches, rats, crane flies, mosquitoes, aphids and wasps. Similar to Kellert’s (11) findings, we found that the most disliked animals were mosquitoes and cockroaches, and, similar to Kellert’s (11) findings in the United States and Bjerke and Østdahl’s [26,32] and Bjerke, Østdahl, and Kleiven’s [32] findings in Norway, rats were the least liked mammals. The squirrel was also the most liked mammal in [22]. However, while Kellert reported that most insects aroused negative sentiments [12,35], we found that there was a wide variety in attitudes towards insects and arthropods in general, similar to the study of Shipley and Bixler [62], or to that of [33], who found that, for example, butterflies were liked by people in Japan while moths and hornets were least liked. Our results also show that within higher taxa there are both species that are strongly liked and those that are strongly disliked. For example, cockroaches and mosquitoes were severely disliked, decreasing the overall score of arthropods, while students had very positive attitudes towards honeybees and fireflies (as in 25), which in turn increased the overall score of arthropods. Our study thus emphasizes that in discussions about the type of nature in cities to be preserved or increased, it may be worthwhile to be very specific with respect to the target species to be supported, rather than referring to taxa as a whole, such as birds or insects.

### 4.3. The Relationship between Familiarity and Attitudes

Environmental education works on the premise that increasing knowledge of the environment positively affects the attitudes towards the environment, eventually leading to responsible environmental behaviour [41]. For individual species, there is evidence that increased familiarity with previously unnoticed species due to attending an environmental education programme can result in a higher appreciation of these species [40]. In our study, there was no relationship between the average familiarity of a species and how positively it was rated on average, i.e., the better-known species were not the more liked ones. For example, deer and mosquitoes were both well known in our study, but the first was well-liked while the second was very disliked. Similar results have been found in a study in Slovakia, where children who had pets had a better knowledge of (a few popular) species, but had more negative attitudes towards species regarded as agricultural pests, such as the mouse or potato beetle [58]. In our study, increasing familiarity increased the range of attitudes towards animal species, so that attitudes on average became more extreme, either positively or negatively. Additionally, unfamiliarity with a species among students did not result in generally negative attitudes towards this species but rather in neutral attitudes, indicating that unfamiliarity does not necessarily result in dislike and can also result in indifference.

When animal species were considered individually, there was a clear positive relationship between familiarity and attitudes among people for the majority of species (*N* = 72 species), i.e., if a person was more familiar with that animal, their attitudes were generally more positive (Figure 1). However, even in such cases, a positive correlation did not always imply that attitudes were positive for the highest levels of familiarity. In our study, this was the case for bugs and muskrats. For many species that have, on average, a neutral or lower attitude value (*N* = 21 species), there was also no significant correlation between students’ familiarity and attitudes (13/21 species). Cockroaches and crane flies were the only animals in our study for which there was a significant negative correlation between attitudes and familiarity across people. More positive attitudes were also associated with increased familiarity for the higher taxon level, except for the ‘other invertebrates’, the group that includes slugs, snails and earthworms, where there was no significant correlation (Appendix E, Figure A4). While our study allows no conclusion to be drawn regarding the success of environmental education programmes in changing people’s attitudes, as educating the students was not part of our study, it emphasizes that even though there is a positive relationship between familiarity and attitudes for many animals one should be careful in assuming that this is generally the case.

### 4.4. Caveats

There are a few notable limitations to our investigation. First, we only had one measure for attitudes and familiarity and relied on people self-reporting how much they liked an animal and how familiar they were with it. While there might be multiple types of ‘liking’ an animal (for example, one could like an animal but does not want the species to occur in one’s vicinity), the self-reported scale is still a useful tool to assess the attitudes of a person to an animal species, as it gives a general idea of the attitudes held. Attitudes towards animals are multifaceted, and condensing them into a single score requires respondents to consider various aspects of their attitudes towards them and turn them into a single generalized score. As a general measure of attitudes this is useful, with the caveat that different contexts can give prominence to specific aspects of these attitudes (for example, what respondents know about a certain species or whether they should consider animals in their direct environment). Students assessed themselves to be largely familiar with the species in the survey. Familiarity implies at least some form of awareness of the species based on the name, probably some skills to identify the species on a photograph and perhaps some deeper knowledge. Such familiarity can arise from personal or vicarious experiences with the species, as well as from reading about it or watching a television programme about it.

Additionally, this study only considered the attitudes of students and may not be representative of the wider population. A follow-up study to test the validity of the results found in this study in the wider population would be desirable. Our study was also designed to test the familiarity and attitudes at the species level, and our analysis of the higher taxonomic levels therefore suffers from an unequal representation of species among higher taxa and an unequal taxonomic resolution within the higher taxa. Nevertheless, we find the taxon-level analysis useful as it clearly indicates that species within the same taxon are regarded differently (cf. birds). Finally, there are many more animals present in cities than only those that we included in our questionnaire. From a practical viewpoint, however, the number of animals we could add to the questionnaire was limited, as we did not wish to overwhelm the participants with too many questions. On the other hand, previous studies have not looked into familiarity with animals per se, but rather have looked at the knowledge that people have about them. In that sense, this study cannot be compared to studies such as those carried out by Kellert (11,12, 27), who asked participants questions about the biology of particular species. Our approach is much less detailed but has the advantage of the inclusion of a large number of species in the questionnaire.

## 5. Conclusions and Research Prospects

We studied the familiarity with and attitudes towards a wide range of animals and found strong differentiation in both familiarity and attitudes. While past investigations have mostly focussed on species with a global distribution [12,22,31], our study focussed on local biodiversity, even though the categories of species were partly generic and could also be applied to a global study (e.g., spiders). To our knowledge, there are very few such studies for urban species, and the list of animals investigated is still very limited [26,33]. In our study, we found no overall relationship between how well-known animal species were and to what degree they were appreciated. Attitudes did become more extreme with increasing familiarity, however. Moreover, participants that were more familiar with certain species also mostly liked them more, but this pattern was not universal. Further research should thus investigate different aspects of ‘attitudes’ [28] for a wide range of species.

The reasons underlying people’s attitudes towards animals are complex and not fully understood (cf. 69, 31). Previous research has pointed to a number of human and animal traits that affect a person’s attitudes towards a particular animal species as well as to the societal and personal context in which a person grows up, including previous experience with animals. For example, fear and disgust towards particular animals such as rats, spiders and snakes has been linked to humans’ innate avoidance of illness and infection [63,64]. Whether or not an animal has a connotation of illness depends on both animal traits and how these are perceived by humans [65]. Understanding why humans value many animals differently will go some way in facilitating understanding of current human–nature relationships, and such knowledge would be of much use in the planning of environments where humans and animals are anticipated to interact.

## Data Availability

Data are available for import via: https://www.soscisurvey.de/stadttiere/?act=i697OE0XILInGEPFW4pyqV8U (accessed on 13 October 2022). R script for import: https://www.soscisurvey.de/stadttiere/?act=i697OE0XILInGEPFW4pyqV8U&rScript (accessed on 13 October 2022).

## Appendix A

### List of Species

We chose these species based on a pilot study that was conducted at the University of Jena. The boundary condition was that more than 100 species would be too many for a questionnaire. We deliberately kept some species that many people may not know but which are sometimes closely associated with human housing (e.g., dormice). Some taxa that were difficult to distinguish for non-experts due to similarity or small size were grouped at the genus level (e.g., redstarts and dormice) or at an even higher taxonomic level (e.g., spiders).

**Table A1:** List of species.

Arthropods	Birds	Mammals	Other Invertebrates	Herpetofauna
Ants	Birds of prey	Badgers	Earthworms	Blindworms
Aphids	Black- and Common redstart	Bats	Slugs	Common adders
Bugs	Blackbirds	Beaver	Snails	Fire salamanders
Bumblebees	City pigeons	Coypu		Frogs
Butterflies	Crows	Deer		Grass- and Smooth snakes
Centipedes	Cuckoos	Dormice		Lizards
Cicadas	Ducks	Edible- and Garden dormouse		Newts
Cockroaches	Greylag- and Field geese	Eurasian otter		Toads
Crane flies	Herons	European hares		
Dragonflies	House- and Field sparrows	Foxes		
Earwigs	Jackdaws	Hedgehogs		
Fireflies	Jay	House cats		
Flies	Kestrel	Mice		
Grasshoppers and Crickets	Kingfishers	Moles		
Ground beetles	Larks	Muskrats		
Honeybees	Magpies	Racoon		
Hornets	Mute swan	Rats		
Ladybugs	Nightingale	Shrews		
Longhorn beetles	Other songbirds (e.g., greenfinch)	Squirrel		
Mosquitoes	Owls	Stone- and Pine marten		
Moths	Pheasants	Weasel, Polecat, and Ermine		
Rhinoceros beetles	Ravens	Wild boars		
Spiders	Rose-ringed parakeet	Wild rabbits		
Wasps	Seagulls			
Wild bees	Starlings			
Woodlice	Storks			
	Swallows			
	Swifts			
	Tits (e.g., Blue Tit, Great Tit)			
	Wood pigeons, pigeons			
	Woodpeckers			

## Appendix B

### Appendix B.1. Data Filtering

The level “other” (*N* = 3) in the factor “gender” was dropped, because there were too few participants in this level for further analysis. The study courses of the students were, where possible, condensed into two groups: (1) “Biology and Environment”, and (2) “Other”. Students with unclear answers to the question about their study course and students that did not fill in their study course were given the study course label “unknown”. The participants were further divided into groups of German nationals and non-Germans. Only students (*N* = 526) were included in the analysis because the other groups of participants in the survey, mostly recruited through social media, were too few in number and too heterogeneous for analysis. Students that were younger than 18 years old (*N* = 1), students with an unknown study course (*N* = 35) and students that were older than 28 years old (*N* = 15) were removed from the dataset due to small sample sizes of age ranges that were not between 18 and 28.

## Appendix C

### Table with Values for Familiarity and Attitudes for Each Species

Familiarity (mean and SD-standard deviation), attitude (mean and SD) and the correlation between familiarity and attitudes for each of the 91 animal species in the questionnaire. Bold values indicate a significant difference from the neutral value after Bonferroni correction. For correlation coefficients, bold values indicate a significant correlation. Animals were sorted from highest familiarity score to lowest familiarity score. *N* = 475.

**Table A2:** Values for Familiarity and Attitudes and the correlation coefficient between them for each species.

Species	Familiarity		Attitude		Correlation Coefficient		Species	Familiarity		Attitude		Correlation Coefficient
	Mean	SD	Mean	SD				Mean	SD	Mean	SD	
Ants	1.97	0.18	2.14	1.16	−0.01		Jay	1.49	0.74	2.99	0.98	0.60
Aphids	1.81	0.46	1.06	1.00	−0.05		Kestrel	1.67	0.54	3.26	0.90	0.41
Badgers	1.89	0.33	2.93	1.03	0.21		Kingfishers	1.65	0.63	3.32	0.91	0.63
Bats	1.96	0.19	3.18	1.03	0.10		Ladybugs	1.98	0.16	3.45	0.83	0.09
Beaver	1.96	0.20	3.31	0.91	0.15		Larks	1.41	0.65	2.92	0.91	0.44
Birds of prey	1.85	0.38	3.29	0.91	0.34		Lizards	1.93	0.26	3.14	0.99	0.20
Black- and Common redstart	1.22	0.82	2.84	0.99	0.62		Longhorn beetles	1.27	0.74	2.13	1.00	0.21
Blackbirds	1.87	0.37	3.19	0.86	0.37		Magpies	1.80	0.46	2.60	1.03	0.19
Blindworms	1.73	0.52	2.29	1.29	0.24		Mice	1.93	0.25	2.60	1.18	0.07
Bugs	1.78	0.46	1.17	1.13	0.10		Moles	1.95	0.25	2.99	1.06	0.13
Bumblebees	1.97	0.17	3.52	0.87	0.16		Mosquitoes	1.95	0.25	0.25	0.61	−0.07
Butterflies	1.73	0.50	2.96	1.08	0.44		Moths	1.76	0.47	2.33	1.09	0.13
Centipedes	1.93	0.29	1.67	1.15	0.06		Muskrats	1.55	0.66	1.81	1.29	0.11
Cicadas	1.32	0.77	1.96	1.08	0.24		Mute swan	1.55	0.71	2.80	1.02	0.42
City pigeons	1.89	0.33	1.88	1.29	0.07		Newts	1.69	0.55	2.69	1.12	0.32
Cockroaches	1.54	0.65	0.77	0.90	−0.18		Nightingale	1.56	0.56	3.22	0.88	0.41
Common adders	1.60	0.58	2.09	1.29	0.14		Other songbirds (e.g., greenfinch)	1.63	0.54	3.33	0.83	0.44
Coypu	0.90	0.92	2.17	1.00	0.29		Owls	1.96	0.21	3.66	0.69	0.19
Crane flies	1.68	0.64	0.84	1.00	−0.23		Pheasants	1.75	0.51	3.04	0.97	0.45
Crows	1.93	0.26	2.54	1.14	0.12		Racoon	1.93	0.28	2.71	1.25	0.09
Cuckoos	1.78	0.43	3.12	0.95	0.22		Rats	1.96	0.20	1.47	1.33	−0.03
Deer	1.98	0.14	3.64	0.67	0.24		Ravens	1.53	0.71	2.69	1.12	0.38
Dormice	1.59	0.61	3.03	1.00	0.38		Rhinoceros beetle	1.41	0.73	2.36	1.12	0.32
Dragonflies	1.96	0.21	3.22	0.99	0.21		Rose-ringed parakeet	0.97	0.81	2.54	0.95	0.49
Ducks	1.99	0.11	3.28	0.83	0.22		Seagulls	1.96	0.21	2.53	1.15	0.15
Earthworms	1.98	0.18	2.65	1.09	0.11		Shrews	1.85	0.40	2.88	1.10	0.15
Earwigs	1.49	0.75	1.28	1.11	−0.01		Slugs	1.96	0.20	1.15	1.07	−0.06
Edible- and Garden dormouse	1.44	0.68	2.81	1.01	0.49		Snails	1.95	0.24	2.53	1.09	0.15
Eurasian otter	1.77	0.48	3.15	1.01	0.35		Spiders	1.98	0.13	1.64	1.32	0.01
European hares	1.94	0.26	3.47	0.79	0.26		Squirrel	1.99	0.10	3.72	0.59	0.09
Fire salamanders	1.82	0.44	3.14	1.00	0.31		Starlings	1.52	0.68	2.83	0.95	0.45
Fireflies	1.91	0.29	3.59	0.73	0.29		Stone- and Pine marten	1.67	0.56	2.31	1.28	0.11
Flies	1.97	0.18	1.24	0.94	−0.05		Storks	1.94	0.29	3.51	0.82	0.36
Foxes	1.97	0.19	3.37	0.89	0.08		Swallows	1.81	0.45	3.18	0.90	0.37
Frogs	1.94	0.24	2.97	1.01	0.14		Swifts	1.31	0.78	2.83	1.05	0.53
Grass- and Smooth snakes	1.65	0.52	2.21	1.34	0.22		Tits (e.g., Blue Tit, Great Tit)	1.80	0.46	3.31	0.88	0.47
Grasshoppers and crickets	1.96	0.20	2.71	1.11	0.01		Toads	1.90	0.32	2.41	1.13	0.09
Greylag- and Field geese	1.85	0.39	2.86	1.01	0.32		Wasps	1.95	0.22	1.37	1.22	−0.07
Ground beetles	1.30	0.76	2.20	0.96	0.32		Weasel, Polecat, and Ermine	1.60	0.59	2.72	1.06	0.32
Hedgehogs	1.99	0.13	3.73	0.59	0.34		Wild bees	1.78	0.46	3.39	0.99	0.27
Herons	1.78	0.51	3.02	0.99	0.40		Wild boars	1.97	0.19	2.62	1.12	0.11
Honeybees	1.96	0.20	3.52	0.85	0.08		Wild rabbits	1.89	0.33	3.43	0.81	0.25
Hornets	1.91	0.32	1.45	1.36	0.01		Wood pigeons, pigeons	1.61	0.59	2.61	1.12	0.27
House- and Field sparrows	1.49	0.67	2.92	0.94	0.48		Woodlice	1.82	0.45	1.38	1.11	0.05
House cats	1.99	0.13	3.27	1.17	0.08		Woodpeckers	1.92	0.30	3.38	0.90	0.28
Jackdaws	1.20	0.81	2.54	0.98	0.50							

## Appendix E

### Taxon-Level Analyses

In order to be able to conduct taxon-level analyses, the values of familiarity and attitude for all species within a taxon were averaged within each participant to produce a taxon-level value for both familiarity and attitudes.
